# Molecular Pathogenesis of Chronic Myelomonocytic Leukemia and Potential Molecular Targets for Treatment Approaches

**DOI:** 10.3389/fonc.2021.751668

**Published:** 2021-09-30

**Authors:** Klaus Geissler

**Affiliations:** ^1^ Medical School, Sigmund Freud University, Vienna, Austria; ^2^ Department of Internal Medicine V with Hematology, Oncology and Palliative Care, Hospital Hietzing, Vienna, Austria

**Keywords:** CMML, chronic myelomonocytic leukemia, molecular, pathogenesis, targets, treatment

## Abstract

Numerous examples in oncology have shown that better understanding the pathophysiology of a malignancy may be followed by the development of targeted treatment concepts with higher efficacy and lower toxicity as compared to unspecific treatment. The pathophysiology of chronic myelomonocytic leukemia (CMML) is heterogenous and complex but applying different research technologies have yielded a better and more comprehensive understanding of this disease. At the moment treatment for CMML is largely restricted to the unspecific use of cytotoxic drugs and hypomethylating agents (HMA). Numerous potential molecular targets have been recently detected by preclinical research which may ultimately lead to treatment concepts that will provide meaningful benefits for certain subgroups of patients.

## Highlights

- CMML is a clinically, molecularly and biologically heterogenous disease
- The combination of molecular data, functional *in vitro* findings, and *in vivo* preclinical models provide a comprehensive view of CMML pathogenesis
- Mutations in *TET2* are common initial clonal driver abnormalities in CMML
- *ASXL1* mutations play a major role in the transformation process into AML
- There is a close correlation between growth factor-independent myeloid colony-formation and the presence of RAS-pathway mutations
- RAS-pathway activation is a crucial pathophysiologic process for GM-CSF hypersensitivity, myeloproliferation, progressive disease and transformation into AML
- Numerous molecular targets provide the rationale for individualized treatment concepts in patients with CMML

## Introduction

Although the term chronic myelomonocytic leukemia (CMML) has been used previously, CMML has been officially, based on morphological criteria/phenotype, acknowledged as a specific entity in the FAB classification 1976 (1, 2). It is characterized by leukocytosis with monocytes and granulocytic cells in all stages of development, marked dysmyelopoiesis, a variable course, unresponsiveness to aggressive chemotherapy and an inherent risk of transformation to acute myeloid leukemia (AML) (3). With regard to the presence of myeloproliferation CMML was originally subdivided into myeloproliferative disorder MP-CMML (WBC count >13 x 10^9^/L) versus myelodysplastic syndrome MD-CMML (WBC count ≤13 x 10^9^/L MD-CMML) by the FAB criteria (4). Since CMML is characterized by features of both a MDS and a MPN the World Health Organization (WHO) classification of 2002 assigned CMML to the mixed category MDS/MPN (5). CMML is further subclassified by WHO into three groups based on blast equivalent (blasts plus promonocytes) in peripheral blood (PB) and bone marrow (BM) as follows: CMML-0 if PB <2% and BM <5% blast equivalent, CMML-1 if PB 2-4% or BM 5-9% blast equivalent, and CMML-2 if PB 5-19% or BM 10-19% blast equivalent, and/or Auer rods are present (6). The median survival of reported series is highly variable indicating a significant clinical heterogeneity of the disease (7–12).

## Pathogenesis of CMML

Cancer is a biologically complex disease with characteristics acquired during the course of a multistep development process. In the past many research tools have been applied to better characterize the phenotypic, genotypic and functional features of cancer and to deeper understand the pathophysiology of malignancy with the ultimate goal to identify prognostic and predictive biomarkers, to render diagnosis more precisely and to develop targeted therapeutics for personalized medicine. No single technology is sufficient to consider all aspects of tumor complexity and information from different technologies are required to provide a comprehensive picture of cancer.

### Structural Analysis by Sequencing Studies

In 1987 a mutation within codon 12 of the *NRAS* gene was reported for the first time by Janssen et al. in a patient with CMML in a study investigating molecular alterations of *RAS* genes in a variety of preleukemic disorders and leukemias of myeloid origin (13). Subsequently is has been shown that *RAS* mutations are rare events in BCR/ABL negative chronic myeloid leukemia (CML) but are prevalent in CMML (14). In this study mutations in the *RAS* oncogene were found in 17 of 30 (57%) CMML patients. In the last years the molecular landscape in patients with CMML has been described by several groups. Molecular abnormalities can be seen in >90% of patients with CMML (15) with a marked heterogeneity among CMML patients. A large number of gene mutations in genes encoding epigenetic regulators (*TET2*, *ASXL1*, *EZH2*, *UTX*, *IDH1*, *IDH2*, *DNMT3A*) (9, 16–22) splicing factors (*SF3B1*, *SRSF2*, *ZRSF2*, *U2AF1*) (23, 24), and cytokine signaling molecules (*NRAS*, *KRAS*, *CBL*, *JAK2*, *FLT3*) have been reported (9, 25–29). Mutations in the transcription regulators *RUNX1*, *NPM1*, and *TP53* have also been found in CMML (9, 30, 31). **Table 1** shows the frequencies of gene mutations in 3 different CMML cohorts in which comprehensive molecular analyses has been reported (9, 11, 32). Considering all molecular data reported mutations in *TET2* (~60%), *SRSF2* (~50%), *ASXL1* (~40%) and RAS pathway (~30%) are most common (15) but no molecular aberration is specific of this entity, as they can be detected with different frequencies in other myeloid neoplasms (33).

**Table 1:** Frequencies of molecular aberrations in different CMML cohorts.

Category	Gene	French n = 312	Mayo Clinic n = 175	Austrian n = 222
Epigenetic regulation	*TET2*	58%	46%	67%
	*ASXL1*	40%	47%	24%
	*EZH2*	5%	1%	16%
	*IDH1*	<1%	2%	NA
	*IDH2*	6%	5%	NA
	*DNMT3A*	2%	5%	8%
				
RNA splicing	*SF3B1*	6%	6%	5%
	*SRSF2*	46%	45%	20%
	*ZRSF2*	8%	5%	7%
	*U2AF1*	5%	8%	6%
				
Cytokine signaling	*NRAS*	11%	12%	15%
	*KRAS*	8%	NA	9%
	*CBL*	10%	14%	10%
	*PTPN11*	NA	5%	5%
	*JAK2*	8%	4%	13%
	*FLT3*	3%	1%	NA
				
Others	*RUNX1*	15%	14%	9%
	*NPM1*	1%	3%	NA
	*TP53*	1%	5%	3%
	*SETBP1*	NA	19%	21%
	*CEBPA*	NA	6%	NA
				

NA, not available.

### Functional Analysis by *In Vitro* Studies

**Figure 1:**
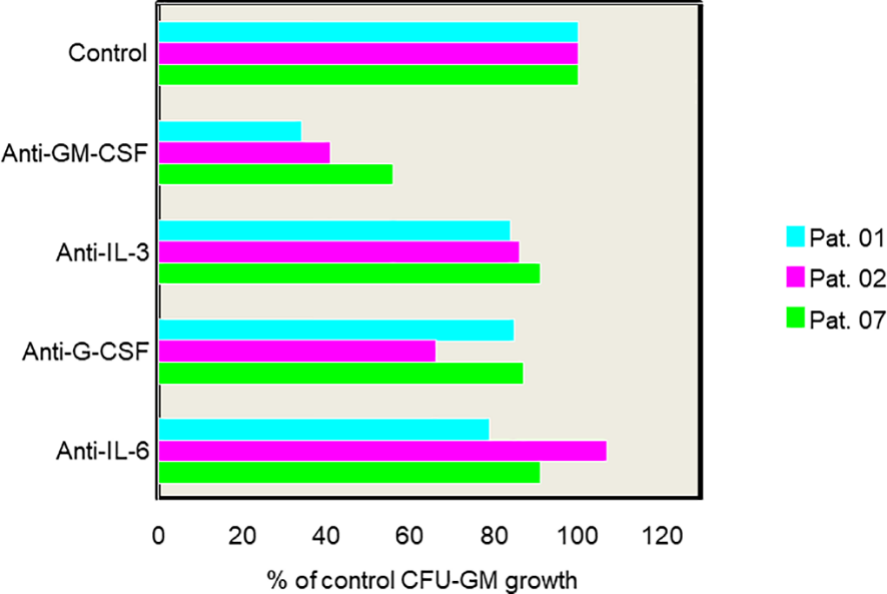
Effect of anticytokine antibodies on spontaneous growth of CMML cells in 3 patients. PB MNC were cultured with medium alone or with antibodies against GM-CSF, G-CSF, IL-3, or IL-6, respectively. Data show a marked inhibition of spontaneous CFU-GM growth in the presence of anti-GM-CSF antibodies in all 3 patients indicating that autonomous colony formation is a GM-CSF dependent *in vitro* phenomenon [adapted from Geissler, K., et al., J Exp Med 1996 (35)].

In 1988 Geissler et al. have originally reported extensive *in vitro* formation of myelomonocytic colony forming units (CFU-GM) without addition of exogenous growth factors in a subset of patients with CMML (**Table 2**) (34). This spontaneous CFU-GM colony formation in CMML was markedly reduced by addition of anti-granulocyte/macrophage colony-stimulating factor (GM-CSF) antibodies, but not by antibodies against other growth factors, suggesting that this is a GM-CSF-dependent *in vitro* phenomenon (35) **Figure 1**. The biologic basis for this observation was later provided by Padron when he reported hypersensitivity of CMML progenitors using phospho-STAT5 flow cytometry (36). Moreover, the group in Vienna could show in a small retrospective study that CMML patients with high spontaneous CFU-GM growth (>100/10^5^ PB mononuclear cells) have an inferior prognosis as compared to patients with low myeloid colony formation suggesting a clinical significance of the original observation (37). These results have been recently extended in a much larger CMML patient cohort indicating that spontaneous myeloid colony-formation was, compared to other single established prognostic factors, the strongest predictor regarding overall survival (OS) (38). This may indicate that *in vitro* cultures using unmanipulated mononuclear cells (MNC) may be a more global test that covers different aspects of malignancy better than any of the single parameters that are currently used to characterize the behavior of a tumor.

**Table 2:** Myeloid colony formation in patients with CMML.

	Source	CFU-C/2.5 x 10^4^ MNC
With CSA	P1 1 BM MNC	910
	6 Controls BM MNC	19.8 ± 8.5
	Pt 2 PB MNC	23.0
	6 Controls	0.36 ± 0.15
Without CSA	P1 1 BM MNC	815
	6 Controls BM MNC	0.0 ± 0.0
	Pt 2 PB MNC	27.0
	6 Controls	0.0 ± 0.0

In vitro cultures from patients with CMML using the CFU-C assay. Mononuclear cells from patients and normal individuals were cultivated in semisolid cultures with or without colony-stimulating activity (CSA). Data show in both CMML patients massively increased myeloid colony (CFU-C) growth as compared to controls and also the formation of CFU-C without the addition of exogenous CSA [adapted from Geissler, K., et al., Leuk Res 1988 (34)].

There is also another *in vitro* phenomenon that seems to be characteristic for CMML patients. Semisolid *in vitro* cultures from PBMNC of normal individuals usually contain a higher concentration of erythroid colonies (BFU-E) as compared to myeloid colonies (CFU-GM). Skewed differentiation toward the myelomonocytic over erythroid commitment in patients, as indicated by an inverse BFU-E/CFU-GM ratio, is a common finding in CMML patients (39). Interestingly, the lack of myelomonocytic skewing separated patients with a particularly favorable prognosis and a minimal risk of transformation.

### *In Vivo* Analysis by Preclinical Mouse Models

Myelomonocytic leukemias can be recapitulated by transplantation of mouse BM cells carrying an oncogenic mutation in the *Nras* locus (40–42). Interestingly, alterations of the other RASopathy genes including *Kras, Cbl, Ptpn11* and *Nf1* may also result in a similar phenotype in preclinical mouse models (**Table 3**) (44–47). In all these *in vivo*-models animals develop a myeloproliferative disorder with clonal expansion of the granulomonopoiesis.

**Table 3:** Mouse models with CMML-like phenotype.

Genotype	Strain	Activation	Phenotype	Reference
*Nras G12D*	C57BL/6	Conditional	Monocytosis, granulocytosis,	Wang (40)
		activation	splenomegaly	
			spontaneous CFU-GM growth	
*Nras G12D*	C57Bl/6	Conditional	Leukocytosis, splenomegaly	Li (41)
		activation	spontaneous CFU-GM growth	
*Nras G12D*	BALB/c	Transgenic	Granulocytosis, monocytosis, mastocytosis	Parikh (42)
		activation	splenomegaly	
*Kras G12D*	C57BL/6	Conditional	Leukocytosis, myeloid hyperplasi a in BM	Chan (43)
		activation	splenomegaly	
			spontaneous CFU-GM growth	
*Kras G12D*	C57BL/6	Conditional	Expansion of progenitor cells in spleen	VanMeter (44)
		activation	spontaneous CFU-GM growth	
*c*-CBL -/-	C57BL/6	Transgenic	Splenomegaly, thrombocytosis	Murphy (45)
		inactivation	lymphoid hyperplasia	
			altered T-cell receptor expression	
*NF1 -/-*	C57Bl/6	Conditional	Leukocytosis, splenomegaly	Le (46)
		inactivation	spontaneous CFU-GM growth	
*PTPN11 D61Y*	C57Bl/6	Conditional	Leukocytosis, anemia,	Chan (47)
		activation	hepatosplenomegaly	
			spontaneous CFU-GM growth	
*TET2 -/-*	C57BL/6	Conditional	monocytosis	Moran-Crusio (48)
		inactivation	splenomegaly	
*TET2 -/-*	C57BL/6	Conditional	Monocytosis, splenomegaly	Li (49)
		inactivation	skewed differentiation toward G/M lineage	
*ASXL1 +/-*	B6.SJL	Conditional	Dyshematopoiesis, leukocytes heterogenous,	Wang (50)
		inactivation	anemia, thrombocytopenia	
			skewed differentiation toward G/M lineage	

The effects of molecular aberrations in genes of the epigenetig machinery have been also studied in preclinical animal models (48–50). In a mouse model with complete functional deletion of *Tet2* resulted in a progressive enlargement of the hematopoietic stem cell compartment and eventual myeloproliferation *in vivo*. *Tet2 +/-* mice displayed increased stem cell self-renewal and extramedullary hematopoiesis, indicating that Tet2 haploinsufficiency contributes to hematopoietic transformation *in vivo* (48). Importantly, one third of *Tet2 -/-* and 8% of *Tet2 +/-* mice died within 1 year of age because of the development of myeloid malignancies reminiscent of CMML indicating that Tet2 loss may represent a predisposition for the development of this malignancy. Moreover, it was shown that transplantation of *Tet2 -/-*, but not wild-type (WT) or *Tet2 +/-* BM cells, was associated with elevated white blood cell (WBC) counts, monocytosis and splenomegaly in WT recipient mice (49).

### Comprehensive View of Pathogenesis

Recent evidence suggests that considering cancer only as a consequence of genetic aberrations is too simple (77). There is growing evidence that the complex nature of transformation from a normal to a cancer cell within different tissues is a result of the interplay among genetic and epigenetic events, tissue structure, exposure and the tissue microenvironment. Thus, molecular analysis of a tumor by NGS alone may be not sufficient to cover the biology of a tumor and emphasize the need for more comprehensive methods to characterize the biology of a tumour. By combining structural data, functional *in vitro* findings, an *in vivo* preclinical models a comprehensive view of pathogenesis of CMML is possible.

**Figure 2:**
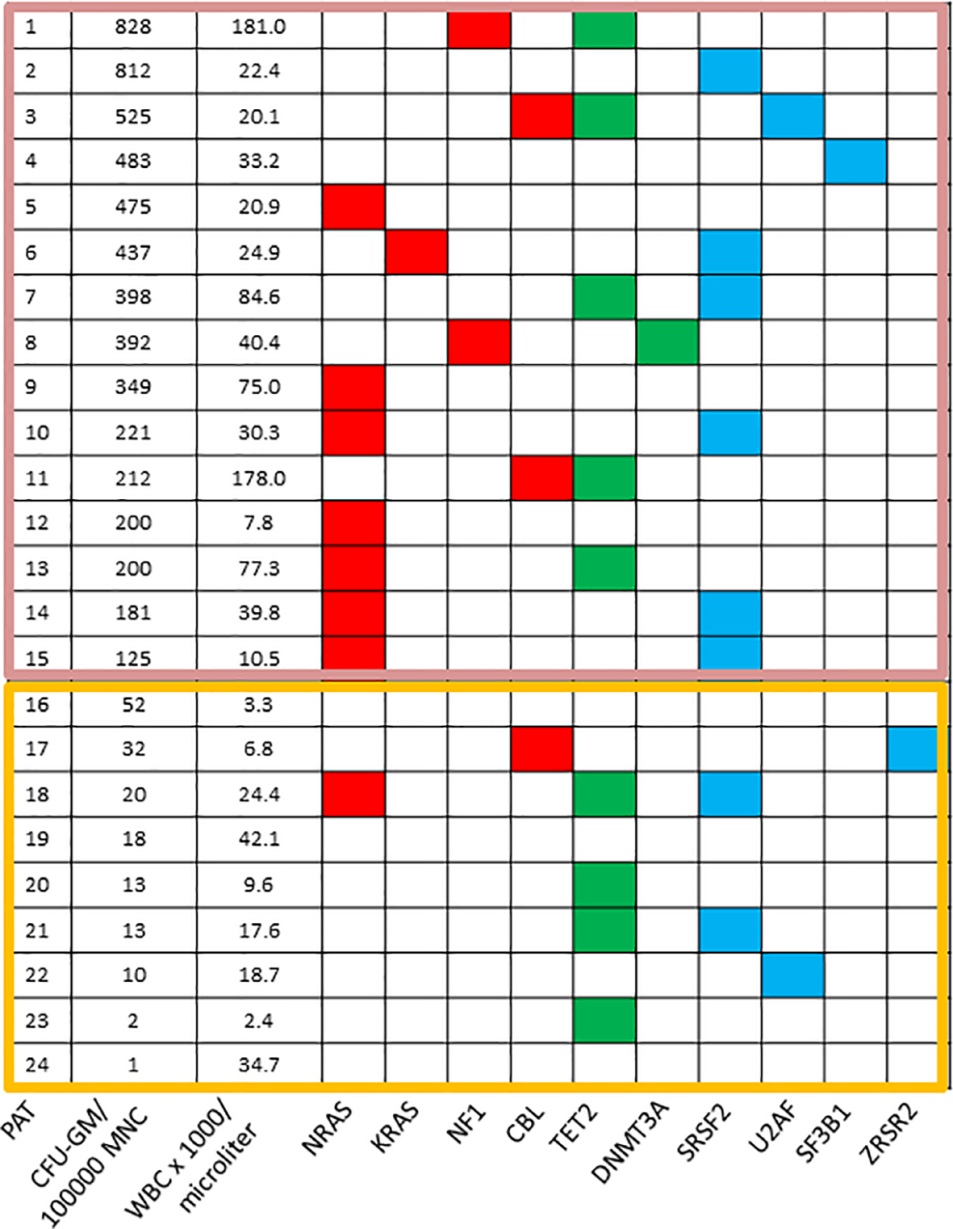
Mutation profiles in CMML according to spontaneous CFU-GM growth. Each row corresponds to one patient. The first column indicates the patient number, the second the number of CFU-GM per 10^5^ peripheral blood mononuclear cells (PBMNC), the third the white blood cell (WBC) count and all other columns represent the status of genes. Colored squares indicate mutated, white squares wild-type genes. The clores of mutant genes indicate the most affected functional categories. Red, green, and blue indicate RAS-pathway, epigenetic factors, and splicing factors, respectively. Mutations in the components of the RAS-pathway were found in 12/15 (80%) CMML patients with high colony growth (≥100 CFU-GM/10^5^ PBMNC) and in 2/9 (22%) patients with low spontaneous colony formation (<100 CFU-GM/10^5^ PBMNC). [adapted from Geissler, K. MEMO 2016 (81)].

Similar to the *in vitro* phenomenon of spontaneous erythroid colony (78) and megakaryocyte colony formation (79) due to hypersensitivity to growth factors in patients with BCR/ABL negative MPN spontaneous myeloid colony formation seems to be an *in vitro* feature in a subset of patients with CMML. Molecular aberrations of RASopathy genes in murine hematopoietic cells induce growth-factor-independent CFU-GM formation *in vitro* due to hypersensitivity of granulomonocytic precursors to GM-CSF (40, 41, 43, 44, 46, 47). Moreover, in juvenile myelomonocytic leukemia (JMML) in which molecular aberrations are mainly restricted to RASopathy genes autonomous myeloid colony formation due to GM-CSF-specific hypersensitivity is a hallmark feature of disease, which has been included in the diagnostic criteria (80). In a small series of CMML patients who had *in vitro* cultures and molecular analyses Geissler et al. observed a close correlation between high spontaneous myeloid colony growth and the presence of RAS pathway mutations as shown in **Figure 2** (81). This initial observation was later confirmed in a larger patient cohort including 100 CMML patients (82). The median number of spontaneously formed CFU-GM/10^5^ MNC was 147.5 in RAS-mutated patients as compared with 2 in RAS-wildtype patients (p<0.00001). Unstimulated myeloid colony formation in RAS-mutated patients was also much higher than spontaneous formation of CFU-GM in normal individuals (median 4.8/10^5^ PBMNC) which has been reported by this group previously (83). There was no significant difference regarding spontaneous CFU-GM formation in CMML patients with molecular aberrations in genes of epigenetic regulation and RNA-splicing, respectively. High spontaneous myeloid colony formation was also never observed in CMML patients with the *JAK2 V617F* mutation as the only molecular aberration in signaling pathways [Geissler et al., unpublished data]. All these findings, in mouse and human, indicate that hypersensitivity to GM-CSF, as manifested by growth factor-independent CFU-GM growth *in vitro*, is caused by molecular aberrations of the RAS-pathway which may be a major driver in CMML pathogenesis, in particular in MP-CMML. Moreover, it reveals high autonomous CFU-GM growth as a functional surrogate parameter of RAS pathway hyperactivation in CMML.

Myelomonocytic skewing has been proposed as a key phenomenon in the pathophysiology of CMML. In a seminal paper using mutation-specific discrimination analysis of single-cell-derived colonies in 28 patients with CMML, Itzykson et al. could show that the main features of this disease are early clonal dominance, arising at the CD34+/CD34- stage of hematopoiesis, and granulomonocytic differentiation skewing of multipotent and common myeloid progenitors (84). Geissler et al. could demonstrate that myelomonocytic skewing as determined by semisolid cultures can separate subgroups of CMML patients with a different phenotype, a different genotype and a different prognosis (39). The definitive link of this phenomenon to the pathophysiology of CMML comes from animal studies in which hematopoietic cells are genetically manipulated with molecular aberrations that are commonly found in CMML patients. Functional inactivation of *TET2* in cord blood CD34+ cells skews progenitor differentiation toward the granulomonocytic lineage at the expense of lymphoid and erythroid lineages (85). In mice, deletion of *Tet2* results in an increased hematopoietic repopulating capacity with an altered differentiation skewing towards the granulomonocytic lineage (49). Other epigenetic modifiers such as ASXL1 have also been shown to impact skewing of hematopoiesis. *Asxl1 -/-* mice had a reduced hematopoietic stem cell (HSC) pool, and Asxl1 -/- HSCs exhibited decreased hematopoietic repopulating capacity, with skewed cell differentiation favoring granulocytic lineage (50). Furthermore the splicing factors SRSF2 and U2AF1 seem to affect skewing. Mutations in both *SRSF2* and *U2AF1* are associated with abnormal differentiation by skewing granulomonocytic differentiation towards monocytes (86). Collectively, many molecular aberrations that can be found in CMML, induce myelomonocytic skewing in the preclinical mouse model providing the genetic basis for this key finding in patients.

### Age Related Mutations in CMML

Recent molecular analyses of large populations have indicated that somatic mutations in hematopoietic cells leading to clonal expansion are commonly acquired during human aging (87). Clonally restricted hematopiesis is associated with an increased risk of subsequent diagnosis of myeloid neoplasia. As some of the genes frequently mutated in age-related clonal hematopoesis such as *TET2* and *ASXL1* are also commonly mutated in CMML and aged hematopoiesis is characterized by a myelomonocytic differentiation bias it was recently hypothesized that CMML and aged hematopoiesis may be closely related (88). Analyses of the somatic mutation landscape of CMML by whole exome sequencing followed by gene-targeted validation indicated that most CMML patients (71%) had mutations in >2 age-related clonal hematopoiesis (ARCH) genes and 52% had >7 mutations overall (89). A higher mutation burden was associated with inferior survival. Considering age-adjusted population incidence and ARCH mutation rates one may speculate that CMML represents the leukemic conversion of the myelomonocytic-lineage-biased aged hematopoietic system.

There is now increasing evidence that mutations in *TET2* are in fact an initial clonal driver in CMML (88, 90). This view is based on the high frequency (60%) of these mutations in CMML patients (9, 19, 21, 22), the fact that *TET2* mutated clones can be detected in a small fraction of older subjects with clonal, but non-leukemic hematopoiesis (90–93), the competitive advantage of murine and human HSC invalidated for *TET2* (48, 85) and the results of single-cell clonal tracking experiments indicating that a *TET2* mutation, when present, is often the earliest recurrent genetic event in CMML (84). According to data from this study the preferred order of mutational accumulation is *TET2* (or *IDH1/2*) or *ASXL1 (EZH2)* first, followed by molecular aberrations in spliceosome components *(SRSF2, SF3B1, U2AF1, or ZRSR2)*. Mutations in the RAS-signaling pathway seem to be rather late events which induce GM-CSF hypersensitivity and myeloproliferation.

### Progression of CMML and Transformation to AML

Around 20% of CMML patients progress and transform to AML. Although the mechanisms behind are not known in detail, available data suggest that molecular aberrations in chromatin modelling as well in cell signaling may contribute to this process. Among genes of the epigenetic machinery, ASXL1 may have the most important impact on transformation. The ASXL1 gene regulates chromatin by interacting with the polycob-group repressive complex proteins (PRC1 and PRC2). Abdel-Wahab et al. reported that *ASXL1* mutations resulted in loss of PRC2-mediated H3K27 trimethylation (94). In a study by Itzykson et al. in which the prognostic impact of different molecular aberrations in CMML patients was studied, only *ASXL1* mutations retained their significant impact on AML-free survival in the multivariate analysis indicating the major role of this molecular aberration in the transformation process (9). Of these, only nonsense and frameshift ASXL1 mutations have been shown to negatively impact OS. The impact of mutations of RAS-pathway components on progression/transformation of CMML is more complex. The first indication of a potential role of *NRAS* aberrations in CMML evolution has been reported, at the molecular level, by Ricci et al. (95). In this study molecular analyses have been performed in 22 MD-CMML patients and in 18 MP-CMML patients. MP-CMML patients had a higher frequency of *RAS* mutations compared with MD-CMML. In two patients who progressed from MDS-CMML to MP-CMML, allele specific PCR showed low levels of the *RAS* mutations at the time of myelodysplastic disease which became predominant in the myeloproliferative phase, documenting for the first time the expansion of a *RAS* mutated clone in concomitance with CMML evolution. Other studies have confirmed that the MPN phenotye of CMML is a disease phase significantly associated with hyperactivation of the RAS-pathway. In a study reported by the Austrian study group MP-CMML as compared to MD-CMML patients had higher circulating blasts, LDH, RAS-pathway mutations, more often splenomegaly and higher growth-factor-independent myeloid colony growth *in vitro* (12). Recently, genetic differences were assessed between subtypes in 973 molecularly annotated Mayo Clinic-GFM-Austrian CMML patients. In this analysis *NRAS* mutations alone did not reach statistical significance as an independent factor impacting AML-free survival, however, the combined oncogenic RAS-pathway category including *NRAS, KRAS* and *CBL* was statistically significant in a model that only included genetic factors (74). Considering the fact, that spontaneous colony formation in CMML functionally covers the most frequent RASopathy gene mutations (38) these data are in line with findings in a small study which have been previously reported. In this study patients with CMML growth-factor-independent colony formation after transformation was significantly increased as compared to CFU-GM growth before transformation (37). Furthermore, a correlation of RAS-pathway mutations and spontaneous myeloid colony growth with progression and transformation could be demonstrated in a retrospective analysis of 337 CMML patients (96). Moreover, recent preclinical models also suggest that activating *Nras* mutations and somatic loss-of-function mutations in *Tet2* exert cooperating effects and accelerate disease progression (97, 98). Altogether, these findings suggest that oncogenic RAS-pathway activation is a phenomenon associated with the MP-CMML phenotype, progressive disease and with transformation to AML.

## Risk Assessment of CMML

The management of patients with CMML should be based on risk assessment. Several studies have shown that the percentage of PB and BM blasts is the most important factor determining survival (7, 99–104). Genetic alterations including gene mutations (7, 9, 10, 32) and chromosomal aberrations (105–107) further refine prognosis and have been included in different prognostic scoring systems. In the EHA guideline from 2018 five risk stratification systems are recommended (7, 9, 10, 106, 108, 109). Mutations in *ASXL1* are included in all 3 molecularly based scoring systems whereas the molecular CMML-specific prognostic scoring system (CPSS-mol) also includes *NRAS, SETBP1* and *RUNX1* (10). A recent study validating different prognostic models demonstrated comparable performance with significant heterogeneity in predicting outcomes (110).

## Treatment of CMML

Traditionally many cancers have been treated with more or less unspecific treatments such as cytotoxic drugs in the past. In a molecular heterogenous malignancy this may have the advantage that many subclones may be impacted by one drug with the potential to improve symptoms associated with a high tumor mass and potentially improve survival. Unfortunately, these drugs often cause significant side effects due to the fact, that also normal cells from tissues with a high proliferation rate may be affected. Targeted drugs on the other hand may be of interest if they are able to specifically hit a cellular component which is critical for the pathophysiology of disease. Many examples from other cancers have shown that with targeted treatment we can expect higher efficacy and lower toxicity as compared to conventional therapy. Unfortunately, CMML is a clinically and molecularly heterogenous disease with sometimes multiple clones that may be pathophysiologically relevant. Theoretically, targeted treatment might offer clinical benefit only if these subclones contribute to inferior prognosis and/or symptoms in patients. Symptoms in patients with CMML are often the clinical consequence of a high tumor mass. Myeloproliferation in CMML is commonly associated with molecular aberrations in cytokine signaling. In particular, as mentioned before, molecular aberrations in components of the RAS signaling pathway are frequently found in these patients. On the other hand there are molecular markers that predict inferior survival. Targeting these components may have the potential to modify the biology of disease and to delay transformation to AML. For such concepts it would be important to know if targeted treatment, at all, will be beneficial in a complex disease such as CMML. Although this question cannot be answered for patients with CMML at the moment, there is some indication from other malignancies that treatment of subclones could be beneficial in patients. Patel et al. published a small series of patients with BCR/ABL negative MPN and a IDH2 mutation who were treated with the IDH2 inhibitor enasidenib which is approved for the treatment of patients with AML harboring this molecular aberration. Although IDH2 is often subclonal in this disease treatment with this IDH2 inhibitor resulted in clinically meaningful responses in these patients (111).

### Unspecific Targeting of DNA Replication by Cytotoxic Molecules

Etoposide (VP16) is a DNA-damaging molecule by inhibition of topoisomerase. Preliminary reports suggested that etposide could give good results in CMML, with true complete responses in some cases and in improvement rather than worsening of cytopenias (51) (**Table 4**). Hydroxyurea (HU), a potent ribonucleotide reductase inhibitor, acts as an S-phase-specific agent with inhibition of DNA synthesis. In a randomized phase III trial in patients with proliferative CMML, HU was more effective and achieved faster response than cytotoxic chemotherapy with VP16 (52). Interestingly, this study remains up to now the only randomized trial in a pure CMML patient population which demonstrated a survival benefit. Allogeneic stem cell transplantation which is the only curative therapy is rarely feasible because of age and/or comorbidities. While unresponsiveness to aggressive chemotherapy is a characteristic for most CMML patients, there may be subgroups that might benefit from more intensive chemotherapy. Although the presence of an *NPM1* mutation, in contrast to AML patients, is an inferior prognostic parameter in CMML, CMML patients with this molecular aberration have shown relatively high response rates in a retrospective analysis (112).

**Table 4:** Potential molecular targets in CMML.

Target	Preclinical information	Clinical study	Reference
DNA-replication unspecific		Phase I/II, n=10; etoposide (VP16) oral 50 mg	Oscier (51)
		2 x weekly – 100 mg 1 x daily	
		ORR: 70%	
		Phase III trial, n=105;	Wattel (52)
		HU arm: n=53; 1 g/d up to 4 g/d	
		ORR: 60%, md OS 20 mo	
		VP16 arm: n=52; 150 mg/wk up to 600 mg/wk	
		ORR: 36%, md OS 9 mo	
DNA-replication CD123-targeted		Phase II, n=10; tagraxofusp relapsed/refractory 80% (8/10) spleen response (≥50% reduction in splenomegaly)	Patnaik (53)
		3 patients achieved bone marrow CR	
Epigenetic machinery unspecific		Phase III, n=358, MDS including CMML	Fenaux (54)
		AZA 525mg/m^2^ per course	
		ORR 27%, md OS 24.5 mo	
		Conventional care group	
		ORR 5%, OS 15.0 mo	
		Phase III, n=170, MDS including CMML	Kantarjian (55)
		DEC 135 mg/m^2^ per course	
		ORR 17%, md OS 12.1 mo	
		Best supportive care	
		ORR 0%, md OS 7.8 mo	
		Phase II, n=19; DEC 100mg/m^2^ per course,	Aribi (56)
		ORR: 69%, md OS 19 mo	
		Phase II, n=31; DEC 135mg/m^2^ per course,	Wijermans (57)
		ORR: 36%, md OS 15 mo	
		Phase II, n=38; AZA 500-525mg/m^2^ per course	Costa (58)
		ORR. 39%, md OS 12 mo	
		Phase II, n=39; DEC 100mg/m^2^ per course,	Braun (59)
		ORR: 38%, md OS 18 mo	
		Phase II, n=10; AZA 500-525mg/m^2^ per course,	Thorpe (60)
		ORR. 60%, md OS 29 mo	
		Phase II, n=76; AZA 375-525mg/m^2^ per course,	Ades (61)
		ORR. 43%, md OS 29 mo	
		Phase II, n=48; AZA 500-525mg/m^2^ per course,	Pleyer (62)
		ORR. 70%, md OS 27.7 mo	
		Phase II, n=43; DEC 100mg/m^2^ per course,	Santini (63)
		ORR: 47.6%, md OS 17 mo	
Epigenetic machinery TET2-, IDH1-, IDH2-targeted	Treatment with vitamin C mimicked TET2 restoration in a reversible transgenic RNAi mouse model	Phase II, Ascorbic acid + AZA	Preclinical Cimmino (64)
		AML, MDS, MDS/MPN with TET2 mutations	
		NCT03397173	
GM-CSF signaling	growth factor independent *in vitro* myeloid colony formation by CMML cells was inhibited by the addition of Anti-GM-CSF antibodies Demonstration of GM-CSF hypersensitivity of CMML progenitors using phospho-STAT5 flow cytometry	Phase II, n=5, rhIL-10 4-8 mcg/kg/day sc no meaningful effects on the WBC counts, 1/3 patients with skin infiltration markedly improved during IL-10 therapy. Phase I, n= of 15, lenzilumab (anti-GM-CSF) 200-600 mg iv days 1, 15 and day 1 in subsequent cycles ORR of 33.3% 3/5 responders were NRAS mutated 1/10 nonresponders was NRAS mutated	Preclinical Geissler (35) Padron (36) Clinical Pöchlauer (65) Patnaik (66)
FLT3 signaling	Increased FLT3-signaling in an MPN model of mice carrying a mutation in the RING finger domain of c-CBL	Phase I/II, quizartinib (FLT3i) + AZA, MDS, MDS/MPN with FLT3 or CBL mutations	Preclinical Rathinam (67)
		NCT04493138	
RAS pathway signaling	The MEKi PD0325901 induced a rapid and sustained reduction in leukocyte counts, enhanced erythropiesis, prolonged survival, corrected aberrant proliferation and differentiation of BM progenitor cells in a *Kras G12D* mouse model The MEKi PD0325901 induced a durable drop in leukocyte counts, enhanced erythropoietic function and markedly reduced spleen sizes in a *Nf1 -/-* mouse model	Phase II, n=11 (RAS mutated CMML cohort); trametinib (MEKi), 2 mg/day orally ORR 27%, md OS 14.5 mo	Preclinical Lyubynska (68) Chang (69) Clinical Borthakur (70)
JAK signaling	The specific JAK2 inhibitor TG101209 inhibited spontaneous CFU-GM growth *in vitro* in all 10 CMML patients tested	Phase I/II, n=20, ruxolitinib in 5-20 mg twice daily ORR 35% 5/9 spleen response 10/11 symptom response	Preclinical
			Geissler (71) Clinical Padron (72) Preclinical
PI3K signaling	Inhibition of PI3K signaling was effective in *Kras+* and *NF1-* mouse models that show many characteristics of CMML including leukocytosis, anemia and splenomegaly		Akutagawa (73)
Cell cycle machinery	Pharmacologic inhibition of PLK1 was effective in *RAS* mutant patient-derived xenografts	Phase II, CFI-400945 (PLK4 inhibitor) + HMA	Preclinical Carr (74)
		AML, MDS, CMML	
		NCT04730258	
Inflammasome	Kras driven myeloproliferation and cytopenia was reversed by functional inactivation of NLRP3 as well as by therapeutic IL-1-receptor blockade.	Phase II, canakinumab (anti-IL-1ß)	Preclinical Hammershe (75)
		LR-MDS, CMML	
		NCT04239157	
Multiple signaling pathway	Combined inhibition of the MEK and JAK/STAT signaling greatly inhibited human and mouse CMML cell growth *in vitro*, rescued mutant *NrasG12D* expressing HSC function *in vivo*, and promoted long-term survival without evident disease manifestation in animals with RAS-pathway driven MP-CMML		Preclinical Kong (76)

ORR, Overall response rates; include CR, complete remission; PR, partial remission; HI, hematologic improvement.

### Specific Targeting of DNA Replication by Antibody-Drug Conjugates

More targeted treatment with cytotoxic drugs can be expected by more detailed immunophenotypical characterization of surface proteins on CMML stem cells which could be used as potential target structures for antibody-drug conjugates (113). One example is the use of the IL-3 receptor as target structure for tagraxofusp, a CD123-directed cytotoxin consisting of human IL-3 fused to truncated diphtheria toxin. This antibody-drug conjugate has shown impressive activity in blastic plasmacytoid dendritic-cell neoplasm (BPDCN) that overexpresses CD123 (114). In an early clinical trial in patients with relapsed/refractory CMML 80% (8/10) of the patients receiving tagraxofusp showed ≥50% reduction in splenomegaly and three patients achieved bone marrow CR (53).

### Unspecific Targeting of the Epigenetic Machinery by Hypomethylating Agents

It is important to note that the approval of the hypomethylating agents (HMA) azacitidine and decitabine (DEC), respectively, was originally based on MDS studies which included only few patients with CMML. In a phase III clinical multicenter trial of 358 MDS patients including 11 patients with dysplastic CMML the median overall survival was 24.5 months in the azacitidine (AZA) group as compared to 15.0 months in the conventional care group leading to the FDA approval of AZA for this subtype of CMML (54). The approval of decitabine (DEC) for CMML was also based on a phase III clinical trial of 170 patients with MDS, 14 of them with CMML (55). The ORR was significantly higher in the DEC group versus supportive care (17% *vs.* 0%, p < 0.001), but the median OS was not significantly different between the two arms. Additional phase II studies confirmed the efficacy of hypomethylating agents in all subtypes of CMML and, therefore, these agents are considered commonly as standard of care for higher risk CMML (**Table 3**) (56–63). In the largest retrospective study with a pure CMML cohort patients were treated with AZA (n = 56) and DEC (n = 65) (115). The ORRs were 41% by the IWG MDS/MPN response criteria (AZA-56%, DEC-58%), with CR rates of <20% for both agents. No significant differences in response rates were seen between MP-CMML and MD-CMML. Similar findings were reported in a smaller, prospective phase II Italian study, with 43 CMML patients receiving DEC (63). The ORR after 6 cycles was 47.6%, with seven CRs (16.6%), eight marrow responses (19%), one partial response (2.4%) and four hematological improvements (9.5%). After a median follow-up of 51.5 months, median OS was 17 months, with responders having a significantly longer survival than non-responders. Despite some efficacy of HMA in CMML patients one has to keep in mind that this treatment does not alter mutational allele burden and disease biology (116).

Proof of efficacy but greatly variable response with HMA provide the rationale for searching biomarkers that predict response. Differentially methylated regions of DNA have been shown to separate DEC responders from non-responders by Meldi (117). Other predictors for response to HMA treatment were reported by Duchmann et al. (118). In a retrospective analysis of 174 CMML patients treated with HMA multivariate analysis mutations in *ASXL1* predicted lower ORR, and *RUNX1* mutations and *CBL* mutations predicted inferior OS. The combination of *TET2* mutation and *ASXL1* wildtype predicted higher CR and better OS. A multicenter retrospective study including 949 non-selected, consecutive CMML patients investigated whether HMA provide a benefit in subgroups of CMML patients (119). Adjusted median OS for patients treated with HU versus HMA was 15.6 months as compared to 20.7 months; (p=0.0002). In patients with MP-CMML, median OS was 12.6 months as compared to 17.6 months; (p=0.0027) for patients treated with HU versus HMA. HMA were not associated with an OS advantage for patients classified as having lower-risk disease (ie, MD-CMML with <10% blasts, CMML-0, or lower-risk CPSS). Considering all the caveats of a retrospective nonrandomized trial these data suggest HMA as the preferred treatment for patients with higher-risk CMML and those with MP-CMML. A recent European multicenter randomized phase III trial evaluating DEC +/- HU *versus* HU in advanced MP-CMML, however, did not show significant differences in outcome. Although HMA definitively play an important role in the management of CMML patients the need for newer, rationally derived therapies is apparent (120).

### Specific Targeting of the Epigenetic Machinery by IDH Inhibitors and TET2 Modifiers

TET2 enzymes have been shown to provide a homeostatic link between intracellular metabolism and epigenetic gene regulation (121). These evolutionary conserved dioxygenases play a key role in the conversion of 5-methyl-cytosine (5-mC) to 5-hydoxymethyl-cytosine (5-hmC). TET dioxygenases require alpha-ketoglutarate, oxygen, Fe(II), and ascorbate for optimal activity (122). Isocitrate dehydrogenase (IDH) is a key enzyme for cellular respiration in the tricarboxylic acid (TCA) cycle. *IDH* mutations found in malignancies block normal cellular differentiation and promote tumorigenesis *via* the abnormal production of the oncometabolite 2-hydroxy-glutarate (2-hG). Recently, two inhibitors targeting *IDH2* and *IDH1* gene mutations, have become important components in AML management since molecular aberrations of *IDH* genes can be found in 20% of patients AML (123, 124). Although mutations involving *IDH1* and *IDH2* are uncommon in CMML (1% and 5-10%, respectively) IDH1/2 inhibitors are likely to present therapeutic options for these patients.

Loss-of-function mutations in *TET2* occur in around 60% of CMML patients and are considered mutually exclusive with *IDH1/2* mutations. Recently there has been accumulated significant preclinical evidence suggesting that ascorbate can restore dysfunctional TET2 activity. Agatocleous et al. generated mice lacking Gulo, the enzyme responsible for ascorbate synthesis. The resulting phenotype resembled mice carrying a homozygous *Tet2* deletion (48). Indeed, ascorbate-depleted stem and progenitor cells showed decreased levels of 5-hmC, predominantly mediated by reduction of Tet2 function (125). On the other hand treatment with vitamin C mimicked Tet2 restoration in a reversible transgenic RNAi mouse model as described by Cimmino (64). Low ascorbate levels have been demonstrated in a subgroup of patients with hematologic malignancies (126). Although no beneficial effects of vitamin C intake regarding leukemia development have been seen in previous reports, these new preclinical data show that the possible impact of supra-physiological concentrations of vitamin C on leukemogenesis remains an interesting treatment concept, particular in CMML-patients harboring a partial or complete loss of TET2 function. In fact the is a current phase II trial which studies the effect of ascorbate in combination with AZA in patients with newly diagnosed AML, MDS, MDS/MPN with *TET2* mutations (NCT03397173).

### Targeting of GM-CSF Associated Signaling

Geissler et al. have shown that growth factor-independent *in vitro* myeloid colony formation by CMML cells can be inhibited by the addition of anti-GM-CSF antibodies, but not by addition of antibodies against IL-6, Il-3, or G-CSF indicating that GM-CSF signaling may play an important role in the pathophysiology of CMML (35). Because of its cytokine synthesis-inhibiting effects IL-10 was studied on CMML cell growth *in vitro*. The addition of IL-10 revealed a profound and dose dependent inhibitory effect on spontaneous *in vitro* growth of CMML cells (35). It was shown that IL-10 induced suppression of CMML cell proliferation was associated with reduced GM-CSF production by leukemic cells, both at the mRNA and protein level. Therefore it was concluded that the inhibitory effect of IL-10 *in vitro* is most likely through suppression of endogenous GM-CSF release. Based on these findings a small pilot trial was initiated in which five patients with CMML were treated with 4µg/kg/day recombinant human IL-10 sc for 1 month and with 8 µg/kg/day for another month (65). Although no meaningful effects of IL-10 treatment was seen on the WBC counts in any of the five patients, one out of three patients with histologically confirmed skin infiltration markedly improved during IL-10 therapy. IL-10 administration was associated with a decline in lysozyme serum levels, a biomarker of the monocytic cell lineage, and downregulation of CD86 which has been shown to be upregulated by GM-CSF and downregulated by IL-10 *in vitro*. Interestingly, the clinical impact of IL-10 war recently supported by a study in which cytokine profiles were analyzed using cryopreserved PB plasma samples from 215 CMML patients (127). CMML patients with decreased IL-10 expression were found to have a poor OS when compared to CMML patients with increased IL-10 expression (P = 0.017), even when adjusted for other prognostic features including ASXL1.

Lenzilumab is a monoclonal antibody with high affinity for human GM-CSF. Based on data showing that anti-GM-CSF antibodies significantly inhibited the growth factor independent myeloid *in vitro* colony formation from primary CMML patient samples (35) and a study reporting that 90% of primary CMML samples demonstrated GM-CSF-dependent STAT5 hypersensitivity (36) lenzilumab was studied in CMML patients. In this early clinical trial of 15 CMML patients the antibody was well tolerated and effective with a durable ORR of 33.3% (66).

### Targeting of FLT3 Associated Signaling

The clinical management of *FLT3*-mutated AML has been changed by the development of FLT3 inhibitors such as midostaurin and gilteritinib which are now in use in the frontline and relapsed/refractory settings in patients with a *FLT3* mutation (128, 129). *FLT3* aberrations have been reported in 1-3% of CMML patients (9, 32). Although these aberrations are uncommon in CMML FLT3 signaling may also occur in wildtype *FLT3* malignancies. Thus, mice carrying a mutation in the RING finger domain of *c-CBL* develop a myeloproliferative disease involving hematopoietic progenitors that show increased FLT3 signaling (67). The incidence of molecular aberrations of the *CBL* gene has been reported from 10-14% (9, 11, 32) and thus is more common than that of the *FLT3* gene. Therefore, CMML patients with mutations in the *CBL* gene could be potential candidates for studies with FLT3 inhibitors. In an ongoing phase I/II trial the FLT3 inhibitor quizartinib in combination with AZA is investigated in patients with untreated or HMA-refractory MDS, MDS/MPN with *FLT3* or *CBL* mutations (NCT04493138).

### Targeting of RAS-Pathway Signaling

Mutated RAS proteins have been deemed “undruggable” for a long time due to their high affinity for GTP and lack of accessible binding pockets. However, the discovery by Ostrem et al. of compounds that covalently bind to the switch II pocket of *KRAS G12C* provided the rationale for the development of inhibitors suitable for clinical testing (130). At the moment this concept does not play an important role in the treatment concepts for CMML, since the *KRAS G12C* mutation is extremely rare in CMML.

RAS proteins require post-translational farnesylation by the enzyme farnesyltransferase to become functionally active. Therefore, inhibitors of this enzyme have been considered as potential candidates for RAS-pathway inhibition. In a clinical phase III trial 85 patients with newly diagnosed JMML, a RAS pathway driven disease, were enrolled between 2001 and 2006 (131). 47 patients received the FTI tipifarnib alone in a phase II window before proceeding to HSCT. Tipifarnib as a single agent was safe and achieved a response rate of 51%, but failed to reduce relapse rates or improve long-term overall survival in the phase III trial. In a preliminary report of a phase II trial in CMML patients tipifarnib was well tolerated, however, had only limited efficacy (132).

The elucidation of the RAS/MEK/ERK signaling pathway in regulating cell proliferation has stimulated the development of selective MEK inhibitors (MEKi). These molecules represent promising therapies for RAS-driven neoplasias and RASopathies associated with hyperactivated RAS signaling. Preclinically, the MEKi PD0325901 was highly effective in reversing the CMML-like phenotype in a *Kras G12D* and in a *NF1 -/-* mouse model (68, 69). In a phase II study in patients with in Neurofibromatosis 1 (NF1) which is a prototypic RASopathy the MEKi selumetinib resulted in at least 20% reduction in the size of plexiform neurofibromas (pNF) from baseline in 71% of patients and was associated with clinically meaningful improvements (133). On the basis of this clinical benefit, selumetinib received FDA approval for children 2 years of age and older with inoperable, symptomatic pNF. In another phase II trial trametinib, another MEKi, was studied in patients with relapsed/refractory leukemias (70). Cohort 1 included patients with relapsed/refractory AML or high-risk MDS with *NRAS* or *KRAS* mutations, cohort 2 patients with AML, MDS, or CMML with a *RAS* wild-type mutation or an unknown mutation status, and cohort 3 patients with CMML with an *NRAS* or *KRAS* mutation. The recommended dose for trametinib was 2 mg orally daily. The overall response rates for cohorts 1, 2, and 3 were 20%, 3%, and 27%, respectively, with a preferential activity among myeloid malignancies with *RAS* mutations. Repeated cycles of trametinib were well tolerated with manageable or reversible toxicities. Thus, some therapeutic potential of trametinib was demonstrated in myeloid malignancies, particularly in RAS-pathway mutated CMML.

### Targeting of JAK-Stat Signaling

There is some evidence of activity or JAK inhibitors in CMML patients. In a study by Geissler the specific JAK2 inhibitor TG101209 was found to either block or strongly inhibit spontaneous CFU-GM growth *in vitro* in all 10 CMML patients tested (71). Among these 10 patients 6 were tested by NGS and, in 5 of them, RAS-pathway hyperactivation was documented due to mutations in NRAS (n=3) or PTPN11 (n=2), respectively. In a *NRAS*-mutant CMML patient who was treated with the JAK1/2 inhibitor ruxolitinib off label, spleen response and the disappearance of constitutional symptoms was associated with a decrease of autonomous CFU-GM formation *ex vivo*. Thus, therapeutic potential of inhibition of the JAK2/STAT5 pathway by ruxolitinib in CMML is suggested. In a phase I/II clinical trial of ruxolitinib in 20 CMML patients the recommended dose of ruxolitinib was 20 mg twice daily and the ORR of 35%, 5/9 spleen responses, and 10/11 symptom responses were seen (72). Correlative analysis demonstrated a downregulation in inflammatory cytokines and GM-CSF-dependent STAT5 phosphorylation in responders. Further studies are required to demonstrate a potential disease modifying effect of ruxolitinib in CMML.

### Targeting PI3 Kinase Signaling

Biological crosstalk is a phenomenon in which one component of a signal transduction pathway can affects another pathway. Thus, the PI3 Kinase-pathway may be aberrantly activated in CMML without molecular aberration in it. Treatment with inhibitors of this aberrantly activated signaling could have the potential to impact malignant cell growth. Using the class I PIK3 inhibitor pictilisib this approach has been successfully applied in a *Kras G12D* and in a *NF1 -/-* mouse model with a CMML-like phenotype (73). In this models, pictilisib attenuated activation of both PI3K/AKT and RAS/MEK/ERK pathways in primary hematopoietic cells. Several PI3K inhibitors have now received regulatory approval for the treatment of breast cancer and B-cell malignancies suggesting that the treatment concept of PI3K-pathway inhibition comes into the clinic (134, 135). Thus, based on some crosstalk between the RAS-signaling and the PI3K/AKT-pathway PI3K inhibitors could be important molecules for the design of future therapeutic strategies for patients with CMML.

### Targeting the Cell Cycle Machinery

In MP-CMML RAS-pathway mutations are associated with a unique gene expression profile enriched in mitotic kinases including polo-like kinase 1 (*PLK1)* (74) as shown in a study using a multiomics platform and biochemical and molecular analyses. In this study unmutated MLL regulated *PLK1* transcript levels *via* promoter monomethylation of lysine 4 of histone 3. In the preclinical mouse model pharmacologic inhibition of PLK1 was effective in *RAS*-mutant patient-derived xenografts providing the rationale for a new biomarker-driven therapeutic approach in patients with proliferative CMML. Currently the administration of the PLK4 inhibitor CFI-400945 with or without HMA is tested in a phase II trial in patients with relapsed/refractory or untreated AML, MDS, or CMML (NCT04730258).

### Targeting of the Inflammasome

The inflammasome is a multimeric protein complex including NLRP3, NLRC4, AIM2 and NLRP6 that initiates an inflammatory form of cell death (pyroptosis) and triggers the release of proinflammatory cytokines (136). Recently, a functional link between oncogenic *Kras G12D* and inflammasome activation was reported in a preclinical model (75). In this mouse model Kras driven myeloproliferation and cytopenia was reversed by functional inactivation of NLRP3. A similar phenotypic improvement was seen with therapeutic IL-1-receptor blockade. Importantly, Kras activation induced the production of reactive oxygen species suggesting that KRAS not only has an ongogenic driver function but also activates the proinflammatory machinery. These findings open a new therapeutic opportunity for Kras mediated malignancies including CMML. Interestingly, there is a current phase II study, in which the anti-IL1ß inhibitor canakinumab is studied in ESA or HMA-refractory low risk-MDS or CMML (NCT04239157).

### Targeting More Than One Pathway

Given the complexity of CMML one can expect, that combinations of molecules impacting different pathways may yield better efficacy. At least in preclinical models this seems to be true (76). In Nras hyperactive mouse models mimicking MP-CMML inhibition of the MEK-pathway alone was only partially effective to improve disease associated features. Despite MEK inhibitor treatment 60% of *Nras G12D* expressing mice died within 20 weeks and surviving animals continued to retain their MP-CMML phenotype. Combined inhibition of the MEK and JAK/STAT signaling, however, greatly inhibited human and mouse CMML cell growth *in vitro*, rescued mutant *Nras G12D*-expressing HSC function *in vivo*, and promoted long-term survival without evident disease manifestation in animals with RAS-pathway driven MP-CMML. Still much work has to be done to address optimal ways to target these pathways in patients with CMML to improve clinical outcome.

## Author Contributions 

The author confirms being the sole contributor of this work and has approved it for publication.

## Funding

This article was supported by the “Gesellschaft zur Erforschung der Biologie und Therapie von Tumorkrankheiten” – ABCMML-112015”.

## Conflict of Interest

The author declares that the research was conducted in the absence of any commercial or financial relationships that could be constructed as a potential conflict of interest.

## Publisher’s Note

All claims expressed in this article are solely those of the authors and do not necessarily represent those of their affiliated organizations, or those of the publisher, the editors and the reviewers. Any product that may be evaluated in this article, or claim that may be made by its manufacturer, is not guaranteed or endorsed by the publisher.
